# Publisher Correction: Inflammation induces two types of inflammatory dendritic cells in inflamed lymph nodes

**DOI:** 10.1038/s12276-018-0086-1

**Published:** 2018-04-13

**Authors:** Jiyoun Min, Dongchan Yang, Mirang Kim, Keeok Haam, Anji Yoo, Jae-Hoon Choi, Barbara U Schraml, Yong Sung Kim, Dongsup Kim, Suk-Jo Kang

**Affiliations:** 1 Department of Biological Sciences, Korea Advanced Institute of Science and Technology, Daejeon, Korea; 2 Department of Bio and Brain Engineering, Korea Advanced Institute of Science and Technology, Daejeon, Korea; 3 Medical Genomics Research Center, Korea Research Institute of Bioscience and Biotechnology, Daejeon, Korea; 4 Department of Life Science, College of Natural Sciences, Hanyang University, Seoul, Korea; 5 Walter-Brendel-Centre for Experimental Medicine, Klinikum der Universität München, Planegg Martinsried, Germany; 6 Biomedical Center, Ludwig-Maximilians-University, Planegg Martinsried, Germany

**Correction to:** *Experimental & Molecular Medicine* **50**, e458; 10.1038/emm.2017.292; published online 16 March 2018

The original version of this article unfortunately contained an error in the author name Dongchan Yang, which was incorrectly given as Dongchan Yang Sung.

This has now been corrected in both the PDF and HTML versions of the article.

